# Supplementary material for: Visual impairment cell non-autonomously dysregulates brain-wide proteostasis
Source: bioRxiv. 2023 Oct 23:2023.10.19.563166. Preprint. [Version 1] doi: 10.1101/2023.10.19.563166 (PMC10634672; doi:10.1101/2023.10.19.563166)
Supplement: 1 [file NIHPP2023.10.19.563166V1-supplement-1.pdf]

bioRxiv preprint doi: https://doi.org/10.1101/2023.10.19.563166; this version posted October 23, 2023. The copyright holder for this preprint (which was not certified by peer review) is the author/funder, who has granted bioRxiv a license to display the preprint in perpetuity. It is made available under aCC-BY 4.0 International license.

Supplementary Data for

Visual impairment non-autonomously dysregulates brain-wide proteostasis.

Shashank Shekhar[1], Katherine J. Wert[2,3], and Helmut Krämer[1,3,4#]

Affiliations:

[1] Department of Neuroscience, UT Southwestern Medical Center; Dallas, TX.

[2] Department of Ophthalmology, Department of Molecular Biology, UT Southwestern Medical Center; Dallas, TX.

[3] O'Donnell Brain Institute, UT Southwestern Medical Center; Dallas, TX.

[4] Department of Cell Biology, UT Southwestern Medical Center; Dallas, TX.

# Correspondence to:

helmut.kramer@utsouthwestern.edu

bioRxiv preprint doi: https://doi.org/10.1101/2023.10.19.563166; this version posted October 23, 2023. The copyright holder for this preprint (which was not certified by peer review) is the author/funder, who has granted bioRxiv a license to display the preprint in perpetuity. It is made available under aCC-BY 4.0 International license.

Supplemental Figures

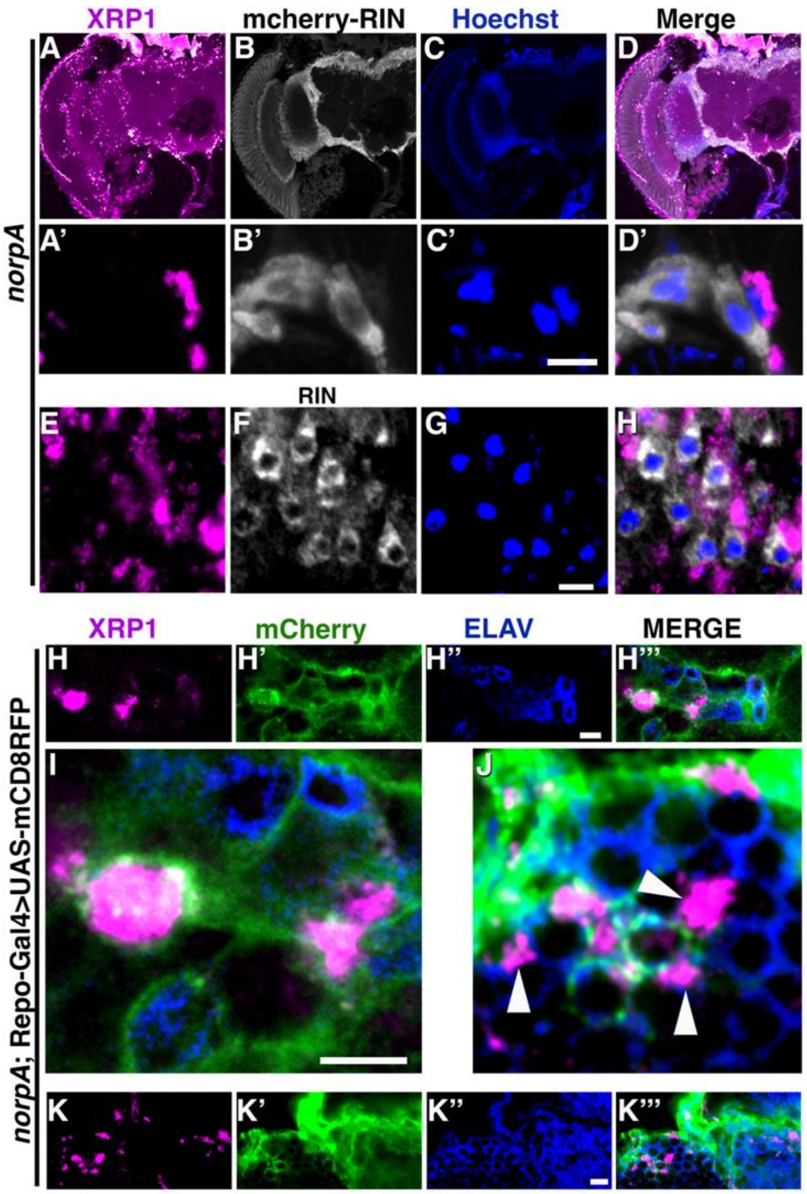

Suppl Fig. 1. Blindness-induced SGs do not colocalize with RIN/G3BP.

(A-D) Cryosections of *norpA;* mCherry-Rin fly brain immunostained for XRP1 (A), mCherry (B) and DNA (C). Merged image shown in (D). (A'-D') High resolution details show exclusion of mCherry-Rin from XRP1-positive SGs.

(E-H) Cryosections of *norpA* head immunostained for XRP1 (E), endogenous RIN protein (F) and DNA (G). Merged image in (H) shows exclusion of Rin protein from XRP1-positive SGs.

(H-K) Cryosections of heads of *norpA* flies expressing UAS-mCD8-RFP under control of the glial repo-Gal4 driver stained for XRP1 (H,K), mCherry (H',K'), and neuronal ELAV protein (H'',K''); merged images (H''',K'''). Magnified images (I,J) show XRP1-positive SGs within glia and neurons (white arrowheads in J).

Scale bars in C', G, and H'' are 5 µm.

Genotypes are listed in Supplementary Table 1.

bioRxiv preprint doi: https://doi.org/10.1101/2023.10.19.563166; this version posted October 23, 2023. The copyright holder for this preprint (which was not certified by peer review) is the author/funder, who has granted bioRxiv a license to display the preprint in perpetuity. It is made available under aCC-BY 4.0 International license.

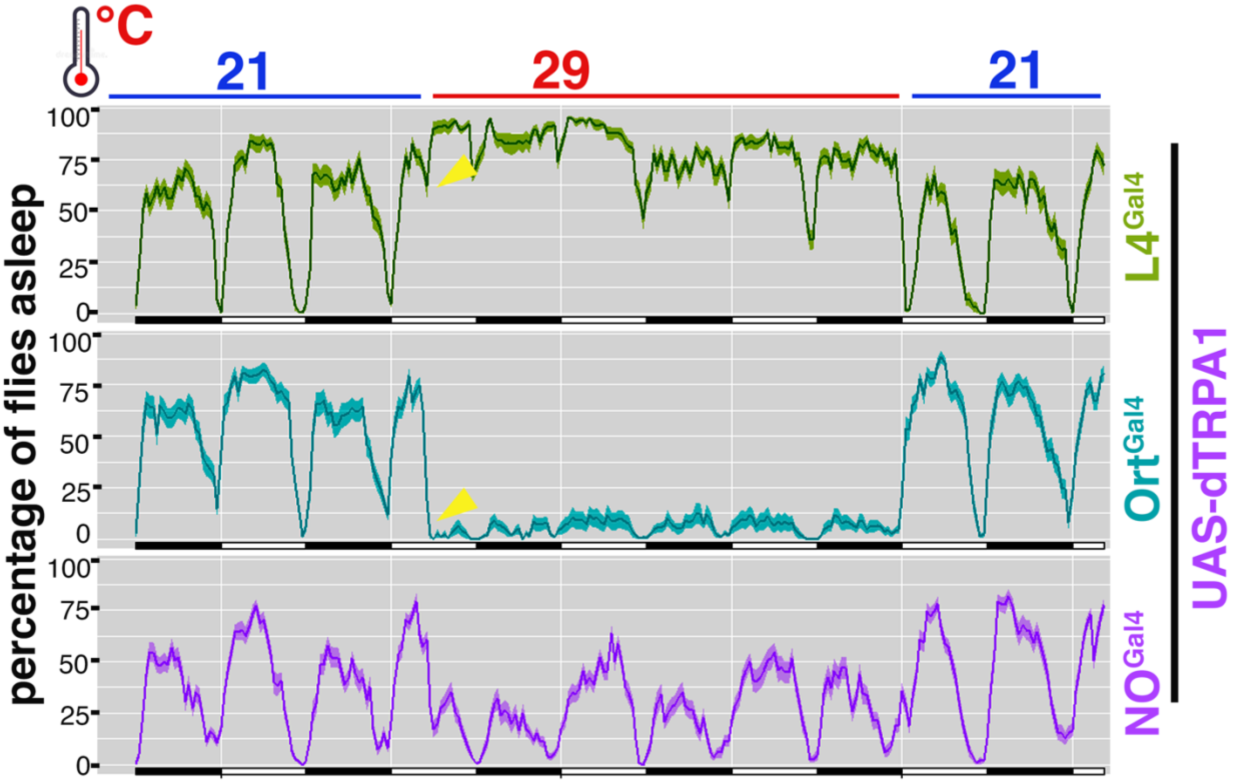

Suppl Fig. 2. Effect of hyperactivation of lamina neuron on fly sleep behavior.

Ethoscope recordings of sleep profile of flies expressing UAS-dTRPA1 driven by L4-Gal4 (green) or ort-GAL4 (Cyan) or without GAL4 (purple), n= 40 per genotype. Restrictive and permissive temperatures are shown on the top, arrow heads indicate the sharp effect on sleep behavior just after switching the temperature. Data shown are combined from two independent experiments with 20 flies each for each genotype.

Genotypes are listed in Supplementary Table 1.

bioRxiv preprint doi: https://doi.org/10.1101/2023.10.19.563166; this version posted October 23, 2023. The copyright holder for this preprint (which was not certified by peer review) is the author/funder, who has granted bioRxiv a license to display the preprint in perpetuity. It is made available under aCC-BY 4.0 International license.

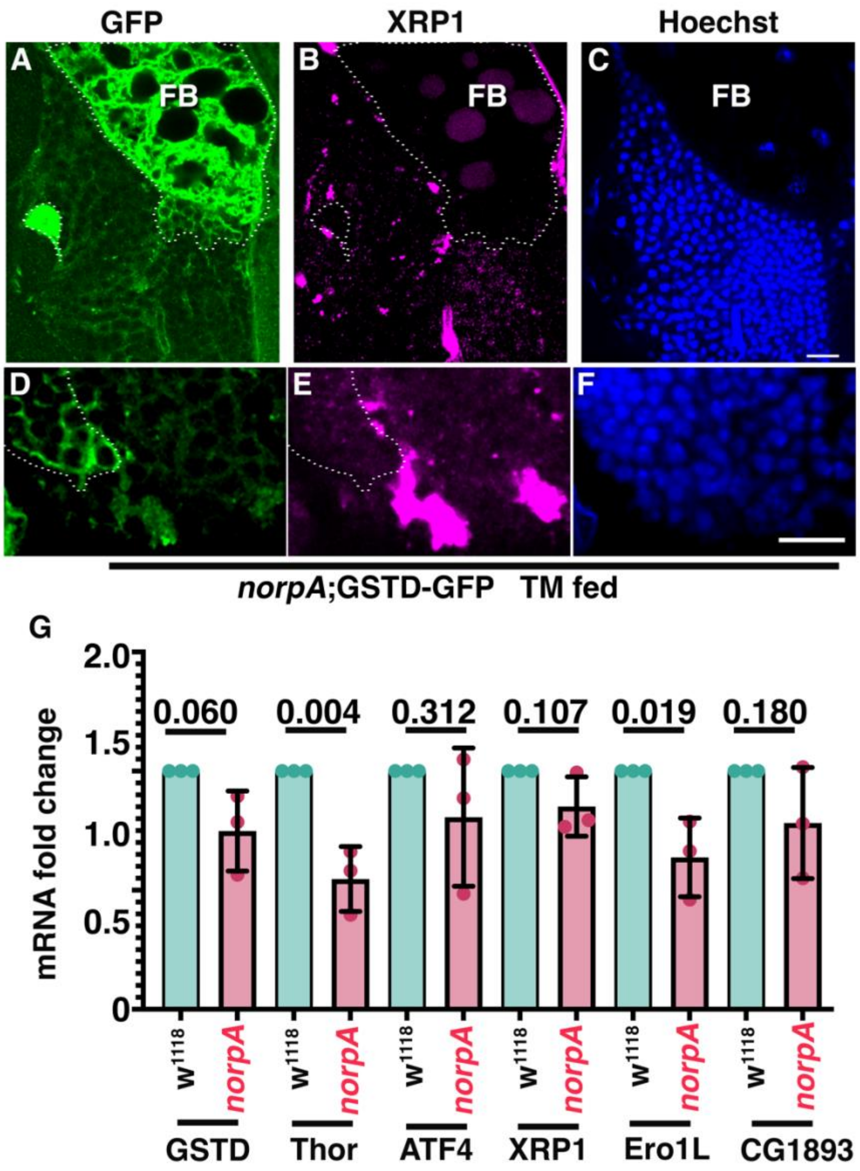

Suppl Fig. 3. Sequestration of ATF4 and XRP1 in stress granules dampens their responsiveness to stress.

(A-F) Adult brain cryosections of TM-fed *norpA* flies expressing GSTD-GFP, a downstream transcriptional target of XRP1, show elevated expression of GSTD-GFP that is limited to cells that lack XRP1-positive SGs (delineated by dotted line). Sections were stained for GFP (A, D), XRP1 (B, E), DNA (C, F). Note the high level of GSTD-GFP expression in fat body cells (FB). Scale bars in C and F are 10 µm.

(G) Bar graphs indicate relative mRNA levels for the indicated ISR-related genes in *norpA* and w^{1118} control flies as determined by RTqPCR. Expression levels were normalized to Act5c RNA for each genotype and to the levels in w^{1118} control flies for each gene.

Genotypes are listed in Supplementary Table 1.

bioRxiv preprint doi: https://doi.org/10.1101/2023.10.19.563166; this version posted October 23, 2023. The copyright holder for this preprint (which was not certified by peer review) is the author/funder, who has granted bioRxiv a license to display the preprint in perpetuity. It is made available under aCC-BY 4.0 International license.

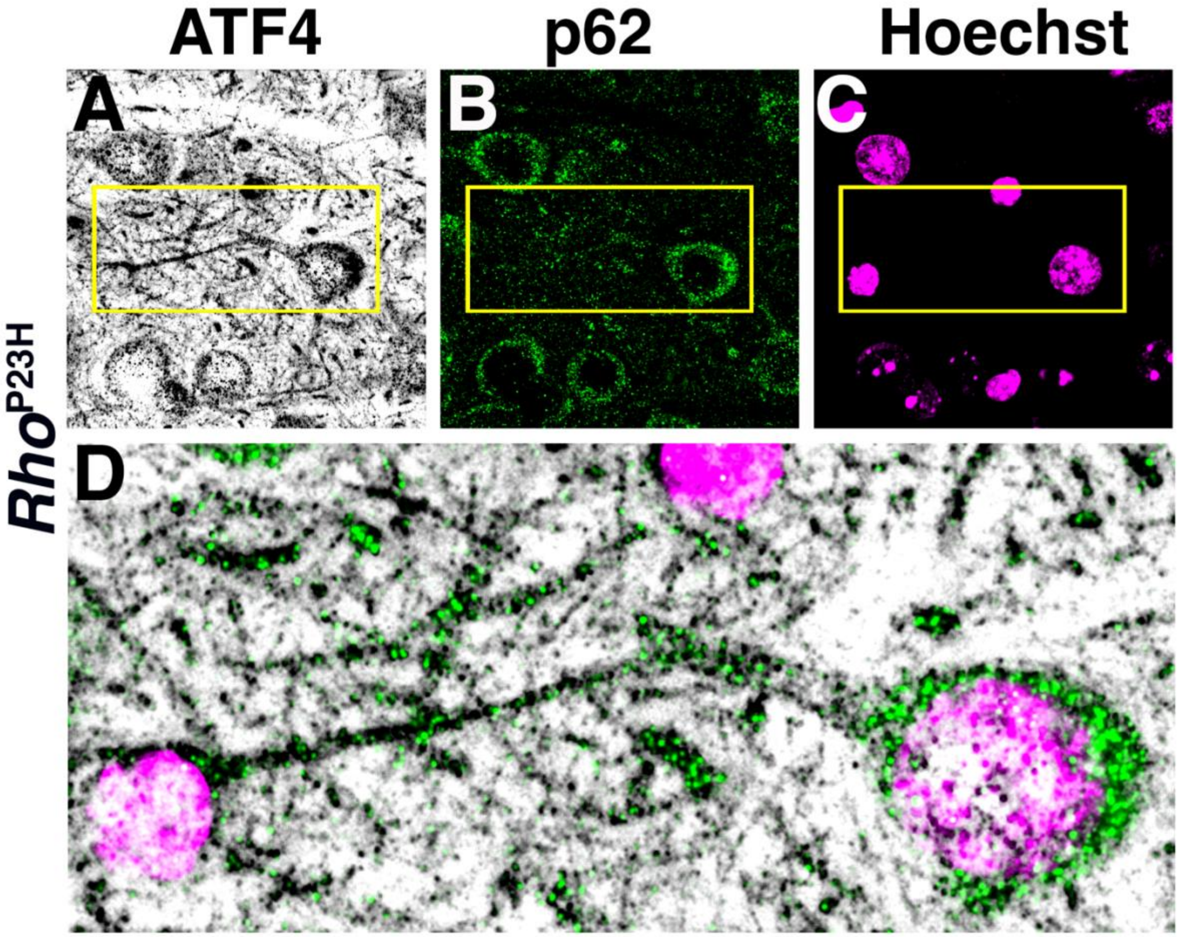

Suppl Fig. 4. Cytosolic puncta of ATF4 and p62 in neurons of *Rho*^{P23H} mice

(A-D) Sections of thalamus from a 10-week-old *Rho*^{P23H} mouse stained for ATF4 (A), p62 (B) and DNA (C). (D) Merged, magnified image of indicated area from A-C shows ATF4 and p62 punctae at high levels in neurites and cell bodies.

Genotype is listed in Supplementary Table 1.

bioRxiv preprint doi: https://doi.org/10.1101/2023.10.19.563166; this version posted October 23, 2023. The copyright holder for this preprint (which was not certified by peer review) is the author/funder, who has granted bioRxiv a license to display the preprint in perpetuity. It is made available under aCC-BY 4.0 International license.

Supplementary Table 1. Genotypes of flies or mice used for each figure

**Figure 1**	**Genotype**
1 B	w^{1118} ;P[w+, QUAS-Shi^{ts1}];
1 C	w^{1118} ; *CarT*$^{HA\text{-}T2a\text{-}QF2}$, P[w+, QUAS-Shi^{ts1}]; *aln*$^{Ty1\text{-}T2a\text{-}Gal4}$
1 D	w^{1118} ; *CarT*$^{HA\text{-}T2a\text{-}QF2}$, P[w+, QUAS-TTL]; *aln*$^{Ty1\text{-}T2a\text{-}Gal4}$
1 H	w^{1118} ;P[w+, QUAS-Shi^{ts1}];
1 I	*BalaT*[ΔGal4]
1 J	*CarT*[43]
**Figure 2**	**Genotype**
2 B, G, I, K	*norpA*[P24]
2 C	*norpA*[P24] ;P[w+=ninaE-norpA.W]2
2 J	w^{1118}
2M-O	*w*[*]; P{w[+mC]=ort-GAL4.C3}10/+;20XUAS-IVS-dTRPA1/+
2P-Q	*w*[1118]; ; 20XUAS-IVS-dTRPA1/P{y+w+GMR31C06-GAL4}^{attP2}10
**Figure 3**	**Genotype**
3 A-C	thor-LacZ
3 D-E	*norpA*[P24]; thor-LacZ
3 G	GSTD-GFP, *norpA*[P24]; GSTD-GFP
3 I	*w*[1118]
3 J	*norpA*[P24]
**Figure 4**	**Genotype**
4 A-I	*Vsx2 and* 129S1/SvlmJ controls
4 J-S	*Rho*^{P23H} *and* C57BL/6J controls

**Suppl. Figures**	
**Suppl. Figure 1**	**Genotype**
A-H	*norpA*[P24]
H-K	*norpA*[P24];; repo-Gal4, UAS-mCD8mCherry
**Suppl. Figure 2**	**Genotype**
top	*w*[*]; p{w[+mC]=L4-Gal4}/+; 20XUAS-IVS-dTRPA1/+
middle	*w*[*]; P{w[+mC]=ort-GAL4.C3}10/+;20XUAS-IVS-dTRPA1/+,
bottom	*w*[*]; ; 20XUAS-IVS-dTRPA1/+,
**Suppl. Figure 3**	**Genotype**
3A-F	*norpA*[P24]; GSTD-GFP

bioRxiv preprint doi: https://doi.org/10.1101/2023.10.19.563166; this version posted October 23, 2023. The copyright holder for this preprint (which was not certified by peer review) is the author/funder, who has granted bioRxiv a license to display the preprint in perpetuity. It is made available under aCC-BY 4.0 International license.

**Suppl. Figure 4**	**Genotype**
A-D	*Rho*^{P23H}
